# Preclinical models of *Bandavirus dabieense* infection: insights into pathogenesis, host responses, and translational applications

**DOI:** 10.3389/fcimb.2026.2007854

**Published:** 2026-10-29

**Authors:** Wan Peng, Ruihua Zhang, Jingxia Wang, Zhouling Jiang, Zhihai Chen

**Affiliations:** National Key Laboratory of Intelligent Tracking and Forecasting for Infectious Diseases, Beijing Ditan Hospital, Capital Medical University, Beijing, China

**Keywords:** antiviral therapy, *Bandavirus dabieense*, preclinical models, severe fever with thrombocytopenia syndrome, vaccine development, viral pathogenesis

## Abstract

*Bandavirus dabieense* (DBV), the causative agent of severe fever with thrombocytopenia syndrome (SFTS), is an emerging tick-borne pathogen associated with substantial morbidity and mortality. Despite increasing incidence across East and Southeast Asia, the pathogenic mechanisms of DBV infection remain incompletely understood, and no specific antiviral therapies have been approved. Robust preclinical models are therefore essential for elucidating viral pathogenesis and accelerating therapeutic and vaccine development. In this Review, we summarize recent advances in cellular and animal models of DBV infection, including established cell lines, primary cells, interferon-deficient rodents, hamsters, ferrets, companion animals, and non-human primates. We critically evaluate the strengths and limitations of these systems in recapitulating key features of human disease and discuss their utility for investigating viral replication, host responses, inflammatory injury, and antiviral efficacy. We further highlight major gaps in the current model landscape and propose priorities for improving the physiological relevance and translational value of DBV experimental systems.

## 1 Introduction

*Bandavirus dabieense* (DBV), previously termed severe fever with thrombocytopenia syndrome virus (SFTSV), is a tick-borne zoonotic pathogen first identified in China in 2009 and formally reclassified by the International Committee on Taxonomy of Viruses in 2019 (Kuhn et al., 2020). Infection with DBV results in severe fever with thrombocytopenia syndrome (SFTS), a disease characterized by fever, thrombocytopenia, leukopenia, gastrointestinal symptoms, and multi-organ dysfunction. Severe cases may progress to neurological complications, shock, and death. Since its identification, DBV infection has been reported in multiple countries, including South Korea, Japan, Vietnam, and Thailand, with case fatality rates varying across outbreaks and study cohorts (Yu et al., 2011; Chang and Woo, 2013; Takahashi et al., 2014; Tran et al., 2019; Rattanakomol et al., 2022). Despite its clinical significance, the mechanisms underlying DBV-induced pathogenesis remain poorly defined, and effective therapeutic options are lacking. The development of reliable experimental models is therefore critical for understanding disease mechanisms and advancing antiviral and vaccine strategies. This review summarizes current cellular and animal models of DBV infection and evaluates their translational relevance.

## 2 Methods

### 2.1 Review design and literature search strategy

This article was designed as a critical narrative review rather than a formal systematic review. The initial manuscript was drafted based on the authors’ prior knowledge of the field and a pre-existing literature collection. In response to peer review, a retrospective structured literature search was conducted during revision on October 14, 2026, to enhance reporting transparency, verify coverage of the evidence base, and identify potentially relevant model-focused studies. We searched PubMed/MEDLINE (via PubMed) and the Web of Science Core Collection (All Editions) from database inception to October 14, 2026.

The search strategy paired a virus-related concept with a model-related concept using the AND operator. The virus concept covered *Bandavirus dabieense*, Dabie bandavirus, severe fever with thrombocytopenia syndrome virus, severe fever with thrombocytopenia syndrome phlebovirus, SFTSV, severe fever with thrombocytopenia syndrome, SFTS virus, and SFTS infection. The model-related concept covered cell models and cell lines, including Vero and Vero E6 cells and HUVECs, as well as macrophages, organoids, animal models (mouse, murine, IFNAR, STAT2, hamster, ferret, cat, feline, dog, canine, and macaque, including non-human primates), and broader *in vitro*, *in vivo*, preclinical, and experimental infection terms. In PubMed, all terms were searched in the Title/Abstract fields; in Web of Science, the corresponding Topic (TS) fields were used. Finally, we cross-checked the reference lists of relevant studies and the authors’ pre-existing literature collection to identify model-relevant references that the database search had not retrieved. The complete database-specific search strings are provided in **Supplementary Tables 1**, **2**.

### 2.2 Eligibility criteria and evidence reconciliation

Evidence was included when it could inform the characterization, comparison, limitations, or translational use of cellular and animal models of DBV/SFTSV infection.

Studies were eligible if they reported viral permissiveness or replication, cellular or tissue tropism, disease phenotype, pathogenic mechanisms, host responses, experimental reproducibility, antiviral or immunomodulatory interventions, neutralizing antibodies, or vaccine efficacy in cellular or animal systems. Acceptable experimental systems included established cell lines, primary cells, ex vivo and organoid cultures, wild-type or genetically modified rodents, neonatal animals, humanized mice, hamsters, ferrets, companion animals, and non-human primates. Natural-infection studies in companion animals were retained when they provided direct evidence on phenotypic or translational validity.

We excluded studies primarily focused on human epidemiology, routine clinical diagnosis or prognosis, vector surveillance, tick ecology, taxonomy, or viral sequencing, as well as studies of molecular structure that did not directly inform the characteristics or applications of experimental models. We retained selected human clinical or pathological studies when they provided comparator phenotypes needed to judge clinical validity, and selected foundational methodological papers when they explained intrinsic properties of widely used model systems. Reviews and editorials served mainly for context and reference-list checking, not as principal evidence for model appraisal. Because the structured search was conducted retrospectively to audit and update a narrative review, it was intended to improve the completeness and transparency of the evidence base rather than to constitute a formal systematic review.

### 2.3 Retrospective literature audit

The two database searches yielded 928 records in total, including 464 from PubMed/MEDLINE and 464 from the Web of Science Core Collection. Deduplication, based primarily on DOI and normalized title, removed 429 duplicate records, leaving 499 unique database records. Distinct publication versions of the same study with different DOIs were retained as separate records. Cross-checking of reference lists and the pre-existing manuscript literature collection identified eight additional references used in the model-focused sections that had not been retrieved by the structured database search. The final model-focused synthesis cites 59 references, of which 51 were retrieved through the structured database search and eight were identified through manual reference-list and literature-library reconciliation. **Supplementary Table 3** summarizes the literature-identification and evidence-reconciliation process. Because the present article remains a critical narrative review, these counts are provided to improve transparency and document the retrospective audit of the evidence base; they should not be interpreted as a prospectively conducted systematic-review study-selection flow.

### 2.4 Framework for critical appraisal of preclinical models

To compare heterogeneous experimental systems more consistently, we appraised the cellular and animal models discussed below against seven predefined domains: clinical and phenotypic validity; virological fidelity; immunological validity; reproducibility; feasibility; throughput and scalability; and purpose-specific utility.

**Box 1** defines these domains. We applied them according to the experimental question rather than combining them into a numerical score, because the available studies differ substantially in viral strain, inoculation route and dose, host age and immune status, experimental endpoints, and outcome measurements.

Box 1 **Criteria for the critical appraisal of DBV preclinical models**

Clinical and phenotypic validity.

Concordance with key human SFTS manifestations; for *in vitro* systems, relevance to clinically affected cell or tissue phenotypes.

Virological fidelity.

Susceptibility to infection, viral replication, and cellular or tissue tropism, as well as viremia and systemic dissemination where applicable.

Immunological validity.

Preservation of relevant innate and adaptive responses, including interferon and inflammatory signaling.

Reproducibility.

Consistency of infection or disease phenotypes and key readouts under defined conditions.

Feasibility.

Accessibility, technical complexity, cost, biosafety, ethical burden, and reagent availability.

Throughput and scalability.

Suitability for parallel testing, repeat experimentation, expansion to larger experimental cohorts, and comparative studies.

Purpose-specific utility.

Suitability for studying pathogenesis and host responses, and for evaluating antiviral agents, immunomodulatory therapies, antibody-based interventions, and vaccines.

*Interpretation: Domains are applied qualitatively in relation to the experimental question and are not combined into a numerical score.*

## 3 Biological characteristics of DBV

DBV is an enveloped, negative-sense RNA virus with virions approximately 80 to 100 nm in diameter (Liu et al., 2014; Saijo, 2018). Its tripartite genome consists of large (L), medium (M), and small (S) RNA segments containing four open reading frames, which collectively yield five major viral proteins (Yu et al., 2011). Specifically, the L segment (6368 nt) encodes the RNA-dependent RNA polymerase (RdRp), the central enzyme responsible for viral transcription and genome replication (Guu et al., 2012). The M segment (3378 nt) expresses a glycoprotein precursor that is proteolytically processed into the Gn and Gc envelope glycoproteins; these mediate host cell entry and virion assembly, serving as the primary targets for neutralizing antibodies (Moming et al., 2021; Sun et al., 2025). Finally, the S segment (1744 nt) adopts an ambisense coding strategy to produce two proteins: the nucleoprotein (NP), which is required for viral transcription, replication, and assembly, and the non-structural protein NSs, a major virulence determinant that antagonizes host innate immune responses (Guu et al., 2012; Lee and Shin, 2021) (**Figure 1**).

**Figure 1:**
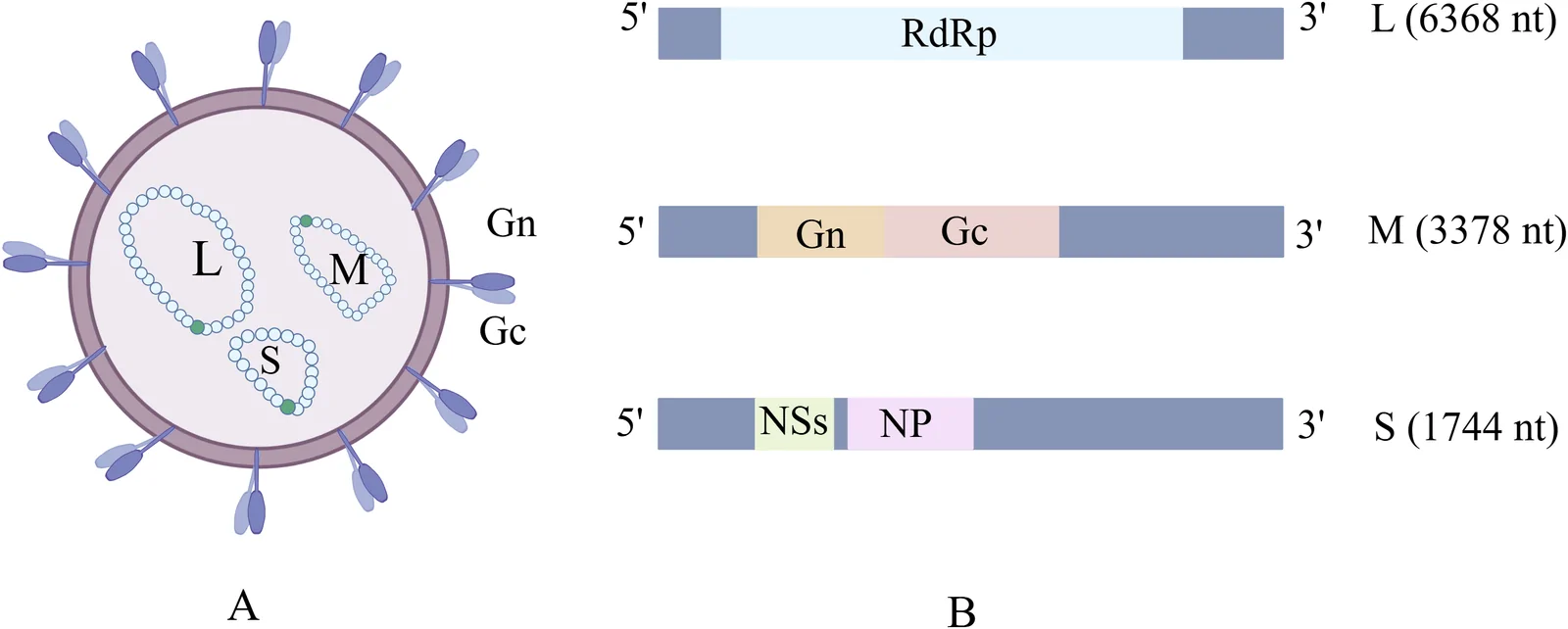
Genome organization and virion structure of *Bandavirus dabieense*. **(A)** Schematic representation of the DBV virion, showing the enveloped particle and the three genomic RNA segments (L, M, and S). The viral envelope contains the surface glycoproteins Gn and Gc. **(B)** Organization of the tripartite RNA genome. The L segment encodes the RNA-dependent RNA polymerase (RdRp); the M segment encodes a glycoprotein precursor that is cleaved into Gn and Gc; and the S segment adopts an ambisense coding strategy to encode the nucleoprotein (NP) and the non-structural protein NSs. Created with BioGDP.com (Jiang et al., 2025).

## 4 Viral tropism, host responses in human DBV infection

### 4.1 Cellular and tissue tropism

DBV exhibits a broad cellular tropism, although the distribution of infected cells varies with disease stage and tissue type. Human pathological studies have identified cells of the mononuclear phagocyte system and B-cell lineage as important targets of infection. In fatal cases, viral antigens have been detected in macrophages and B-lineage cells, particularly plasmablast-like cells, within secondary lymphoid tissues (Suzuki et al., 2020). These findings indicate that lymphoid tissues are important sites of viral infection and suggest that infected immune cells may contribute to systemic viral dissemination.

The spleen and lymph nodes are prominently affected in severe SFTS, with pathological changes including lymphoid depletion and tissue necrosis (Suzuki et al., 2020). Viral antigen-positive cells have also been observed in non-lymphoid organs, although the presence of such cells does not necessarily establish productive infection of the resident parenchymal cells. Liver injury is common, as reflected by elevated serum transaminases, whereas neurological involvement may occur in severe or fatal cases (Liu et al., 2014; Saijo, 2018). Experimental studies further show that DBV can infect endothelial cells and central nervous system cells (Li et al., 2018; Gao et al., 2022). Nevertheless, the relative roles of direct viral replication and secondary immune-mediated injury in organ dysfunction in human SFTS remain incompletely understood.

### 4.2 Host immune responses

The clinical course of DBV infection reflects a balance between antiviral immune responses and virus-induced immune dysregulation. Type I interferon signaling is central to the early antiviral response, yet the viral nonstructural protein NSs disrupts host antiviral signaling to drive immune evasion (Lee and Shin, 2021). Severe infection is frequently accompanied by elevated inflammatory mediators, including IL-6, IL-1β, and other cytokines linked to systemic inflammation (Bryden et al., 2022; Huang et al., 2022).

Impaired adaptive immunity has also been associated with poor outcomes. Fatal SFTS cases may show defective virus-specific antibody responses and disturbed B-cell and T-cell function (Song et al., 2018), defects that can compromise viral clearances and sustain viremia. Thrombocytopenia, leukopenia, coagulation abnormalities, and endothelial injury further characterize severe disease (Li et al., 2018; Saijo, 2018). Together, these observations suggest that disease progression reflects both insufficient antiviral control and excessive inflammation, although the balance between these processes likely varies among patients.

### 4.3 Risk factors for severe disease

Clinical outcomes following DBV infection vary widely. Advanced age is consistently associated with an increased risk of severe disease and mortality (Chen et al., 2017; He et al., 2020), and underlying conditions such as diabetes and hypertension are likewise linked to unfavorable outcomes (Wang et al., 2026). High viral loads, marked thrombocytopenia, coagulation abnormalities, and evidence of organ injury are additional features associated with mortality (Chen et al., 2017; He et al., 2020). These clinical associations should be distinguished from factors that increase exposure risk, such as agricultural activities and tick contact.

The greater severity seen in older patients may reflect age-related changes in antiviral immunity and inflammatory regulation, although the underlying mechanisms remain incompletely defined. The impact of pre-existing immunosuppression on disease outcome also warrants further investigation. These clinical observations underscore the need to consider host age, immune competence, and underlying health status when selecting experimental models. Models that retain functional immune responses may be particularly valuable for studying age-associated susceptibility and inflammatory injury, whereas interferon-deficient models are better suited to examining viral replication and antiviral efficacy.

## 5 *In vitro* cellular models of DBV infection

*In vitro* cellular systems are indispensable for investigating DBV entry, host signaling pathways, and high-throughput antiviral screening. However, severe SFTS involves complex host responses that cannot be fully reproduced in conventional cell cultures. Clinical studies have reported elevated levels of inflammatory mediators, including IL-6, IL-8, IL-10, and IFN-γ, several of which are associated with disease severity (Liu et al., 2017; He et al., 2021). DBV infection also causes vascular endothelial injury, increased endothelial permeability, and disruption of intercellular junctions, each potentially contributing to tissue dysfunction (Li et al., 2018). The involvement of immune cells, vascular endothelial cells, and multiple organs indicates that disease progression cannot be explained by viral replication in a single cell population. Although conventional cell lines are useful for examining specific host-virus interactions, they cannot adequately reproduce the coordinated inflammatory responses and multicellular interactions seen in severe SFTS. Consequently, findings derived from cell-based studies require validation in appropriate animal models to establish their relevance to disease pathogenesis. Currently, both established cell lines and primary cells are employed, depending on the specific experimental context (**Table 1**, **Figure 2**).

**Table 1:** *In vitro* cellular models of *Bandavirus dabieense* infection and their applications in antiviral evaluation.

Model type	Cell line/source	Characteristics	Applications	Representative agents/targets	Reference
Established cell lines	Vero/Vero E6	Deficient in type I interferon production; highly permissive to viral replication	Virus isolation and propagation; high-throughput antiviral screening; cytopathic effect analysis	Favipiravir, ribavirin; bergamottin, hexachlorophene	(Desmyter et al., 1968; Osada et al., 2014; Tani et al., 2016; Lee et al., 2017; Yuan et al., 2019; Anggita et al., 2025; Lokupathirage et al., 2025; Mekata et al., 2025; Wan et al., 2025)
	HepG2/Huh-7	Human hepatocyte-derived; retain metabolic enzyme activity; permissive to infection	Modeling liver injury; evaluation of hepatotoxicity and prodrug activation	Calcium channel blockers (e.g., benidipine); lipid metabolism inhibitors	(Li et al., 2019; Liu et al., 2025; Wu et al., 2025)
	THP-1-derived macrophages	PMA-differentiated; capable of M1/M2 polarization	Investigating macrophage-associated inflammatory responses	Immunomodulatory agents targeting macrophage polarization or inflammasome pathways	(Peng et al., 2016; Zhang et al., 2019; Huang et al., 2022; Jin et al., 2023; Zhang et al., 2024)
Primary cell models	HUVECs	Preserve endothelial barrier integrity and intercellular junctions	Analysis of vascular permeability and endothelial dysfunction	Kinase inhibitors; barrier-stabilizing compounds	(Li et al., 2018; Jin et al., 2025)
	Primary macrophages (human/mouse)	Physiological target cells; support viral replication and persistence	Investigation of viral reservoirs, pyroptosis, and platelet–macrophage interactions	NLRP3 inflammasome inhibitors	(Jin et al., 2012; Song et al., 2018; Yu et al., 2023)

PMA, phorbol 12-myristate 13-acetate; HUVECs, human umbilical vein endothelial cells.

**Figure 2:**
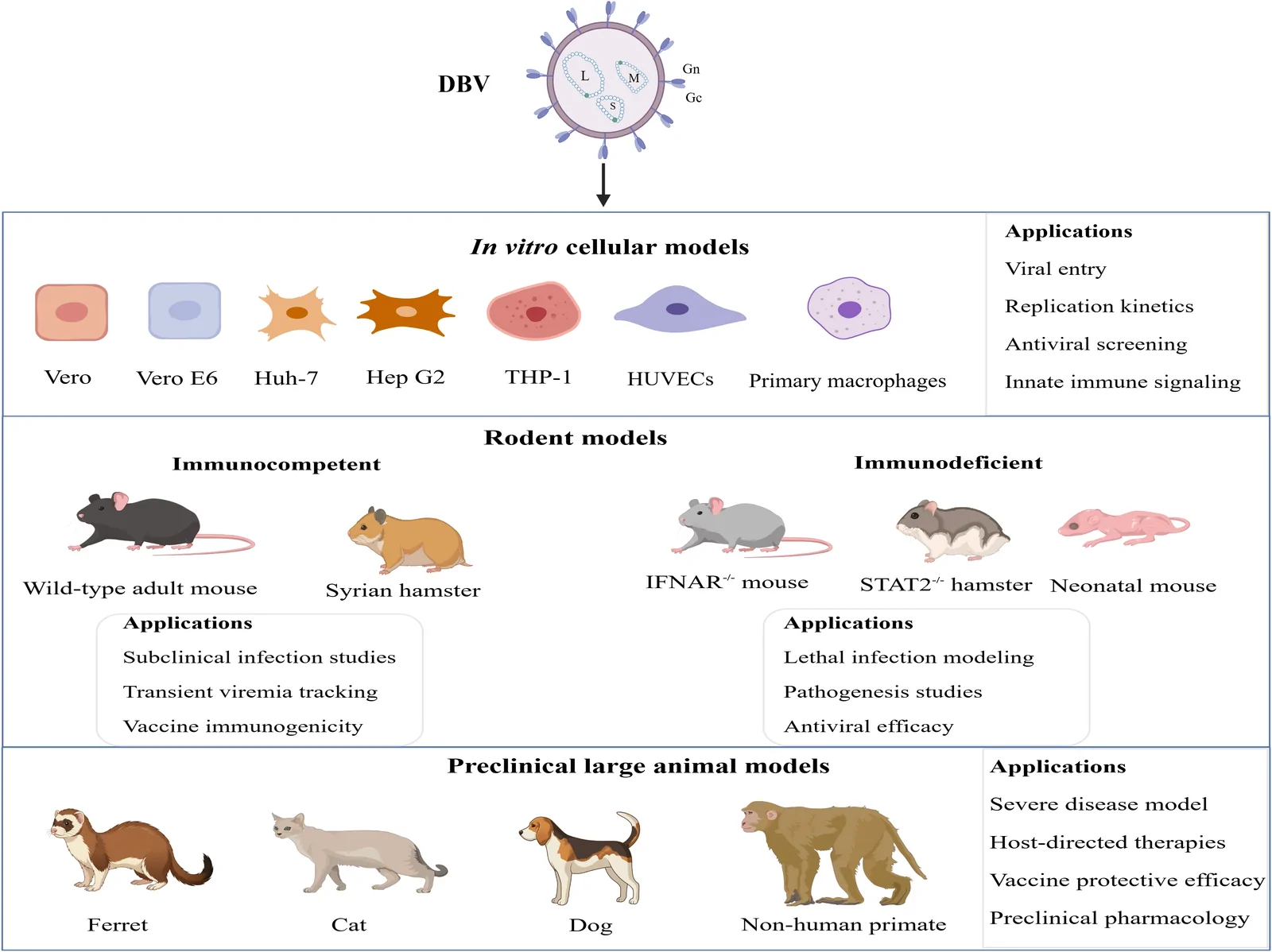
Overview of experimental models for *Bandavirus dabieense* infection. Graphical summary of currently used *in vitro* and *in vivo* models for DBV research. *In vitro* systems include established cell lines (e.g., Vero, Huh-7, Hep G2, THP-1) and primary cells (e.g., HUVECs and macrophages), which are primarily used to investigate viral entry, replication, and host responses. Rodent models comprise immunocompetent animals, which exhibit limited susceptibility, and immunodeficient models (e.g., IFNAR^−^/^−^ mice, STAT2^−^/^−^ hamsters, and neonatal mice), which support severe or lethal infection. Preclinical large animal models, including ferrets, cats, dogs, and non-human primates, provide complementary systems for studying disease severity, host–virus interactions, and therapeutic efficacy. Together, these models capture distinct aspects of DBV pathogenesis and support antiviral and vaccine development. Created with BioGDP.com (Jiang et al., 2025).

### 5.1 Established cell lines

Established cell lines remain the most widely used tools in DBV research due to their ease of manipulation, rapid proliferation, and experimental reproducibility. Vero cells and the Vero E6 subclone, derived from African green monkey kidney epithelium, are routinely used for viral isolation, propagation, and titration (Anggita et al., 2025; Mekata et al., 2025). Owing to their deficiency in type I interferon (IFN-α/β) production, these cells support efficient DBV replication (Desmyter et al., 1968; Osada et al., 2014). In this context, DBV infection induces caspase-dependent cell death in Vero E6 cells (Lokupathirage et al., 2025). These highly permissive systems are also extensively used for antiviral drug screening. Screening of FDA-approved compound libraries in Vero cells has identified ribavirin and favipiravir (T-705) as inhibitors of DBV replication (Tani et al., 2016; Lee et al., 2017). Additional compounds, including viral entry inhibitors and natural products such as bergamottin and hexachlorophene, have likewise been evaluated in this platform (Yuan et al., 2019; Wan et al., 2025).

Human-derived cell lines are frequently used to investigate cell-specific host responses during DBV infection. Hepatocyte-derived lines, including HepG2 and Huh-7, are highly permissive to DBV infection and are widely used to study aspects of virus-associated liver injury, a key clinical feature of SFTS (Liu et al., 2025; Wu et al., 2025). Compared with Vero cells, these systems are particularly suitable for evaluating drug-induced hepatotoxicity and for testing compounds requiring hepatic metabolic activation. For example, studies using Huh-7 cells have demonstrated that calcium channel blockers (e.g., benidipine) and inhibitors of lipid metabolism can suppress DBV replication, highlighting the importance of host metabolic pathways in viral infection (Li et al., 2019). However, these cell lines cannot fully recapitulate the complex tissue-level pathology and systemic inflammatory responses observed during human DBV infection.

Immune cell models are essential for investigating host inflammatory responses. Peripheral blood mononuclear cells (PBMCs) comprise heterogeneous immune cell populations, including monocytes and lymphocytes, and have been used to study immune responses associated with SFTS. For example, transcriptomic analysis of PBMCs isolated from SFTS patients has revealed alterations in immune-related gene expression, including differences between patients with and without encephalitis (Wu et al., 2025). THP-1-derived macrophages are widely used to study virus-induced immune responses (Peng et al., 2016; Jin et al., 2023; Zhang et al., 2024), although they do not reproduce the cellular heterogeneity of PBMCs. DBV infection has been shown to promote polarization toward an M2-like phenotype, which may impair antiviral immunity and facilitate viral persistence. These macrophage-based systems are therefore useful for distinguishing compounds that directly suppress viral replication from those that primarily modulate inflammasome activation, cytokine release, or macrophage polarization (Zhang et al., 2019; Huang et al., 2022).

### 5.2 Primary cell models

Primary cells retain key physiological features derived from their tissue of origin and thus provide a more biologically relevant system for studying DBV pathogenesis. Human umbilical vein endothelial cells (HUVECs) are commonly used to investigate virus-induced vascular dysfunction, a hallmark of hemorrhagic manifestations in SFTS. DBV infection disrupts intercellular junctions and cytoskeletal organization in HUVECs, leading to increased endothelial permeability and barrier dysfunction (Li et al., 2018). This system has also been applied in antiviral evaluation, particularly for identifying agents that preserve vascular integrity, including kinase inhibitors and barrier-stabilizing compounds (Jin et al., 2025).

Primary macrophages derived from human or murine tissues represent another important model, as these cells are key targets of DBV *in vivo* (Song et al., 2018; Yu et al., 2023). Splenic macrophages, in particular, support robust viral replication and may serve as sites of viral persistence (Jin et al., 2012). Beyond their role in viral replication, macrophages are central to the dysregulated inflammatory response associated with severe disease. For example, DBV infection induces macrophage pyroptosis through activation of the NLRP3 inflammasome, whereas platelets have been shown to attenuate this process, highlighting a complex interplay between immune and hemostatic systems during infection (Yu et al., 2023).

## 6 Animal models of DBV infection

SFTS is characterized by a complex, multisystem pathogenesis, necessitating robust *in vivo* models to elucidate the infectious mechanisms of DBV and evaluate candidate therapeutics. As summarized in **Figure 2**, current DBV models span *in vitro* systems, rodent models, and large animal models, each capturing distinct aspects of disease pathogenesis and therapeutic response. However, modeling DBV infection presents significant challenges, as most immunocompetent small animals, such as adult mice and hamsters, typically resist clinical infection (Liu et al., 2014; Matsuno et al., 2017). Consequently, researchers have successfully adapted immunodeficient or specialized systems—including interferon α/β receptor-deficient (IFNAR^-/-^) mice, neonatal mice, STAT2-knockout hamsters, and aged ferrets—to recapitulate severe human disease (Gowen et al., 2017; Park et al., 2019; Bryden et al., 2022; Gao et al., 2022). Furthermore, humanized mice, non-human primates (NHPs), and companion animals provide valuable platforms for more advanced pathogenesis and efficacy studies (Park et al., 2019; Xu et al., 2021; Ishijima et al., 2022; Li et al., 2023) (**Table 2**).

**Table 2:** *In vivo* animal models of *Bandavirus dabieense* infection for pathogenesis studies and preclinical evaluation.

Animal model	Immune status	Disease features	Translational applications	Limitations	Reference
Wild-type adult rodents (e.g., C57BL/6 mice, Syrian hamsters)	Immunocompetent	Resistant to disease; asymptomatic infection; rapid viral clearance; no mortality	Assessment of vaccine immunogenicity (humoral and cellular responses); investigation of innate restriction mechanisms	Lack of clinical disease limits evaluation of antiviral efficacy and protective outcomes	(Liu et al., 2014; Matsuno et al., 2017)
Immunocompromised rodents (e.g., IFNAR^−/−^ mice, STAT2^−/−^ hamsters)	Deficient in type I interferon signaling	Severe and often lethal infection; sustained viremia; multi-organ involvement (liver, spleen)	Standard model for *in vivo* antiviral efficacy testing; pharmacokinetic and dose–response studies	Impaired immune system limits evaluation of host-directed therapies and vaccine-induced protection	(Tani et al., 2018; Bryden et al., 2022; Huang et al., 2022; Shimojima et al., 2022; Hicks et al., 2024; Kim et al., 2024; Xu et al., 2024; Jeong et al., 2025)
Neonatal mice	Immature immune system	Rapid disease progression; high mortality; neurotropic infection with CNS involvement	Studies of neuroinvasion; early-stage evaluation of antivirals and neutralizing antibodies	CNS-dominant pathology differs from human systemic disease; limited utility for immune response studies	(Chen et al., 2022; Gao et al., 2022)
Humanized mice	Immunodeficient NCG mice engrafted with human PBMCs	Thrombocytopenia, leukopenia, cytokine dysregulation, and multi-organ injury	Investigation of human immune responses and evaluation of human-targeted therapeutics	Donor-dependent variability; incomplete immune reconstitution; technical complexity	(Xu et al., 2021)
Ferrets	Immunocompetent	Age-dependent disease: severe infection in aged animals (cytopenia, fever, high mortality); mild disease in young animals	Evaluation of vaccine efficacy and immunomodulatory therapies; modeling age-associated disease severity	High cost; requirement for aged cohorts; sensitivity to inoculation route	(Park et al., 2019; Park et al., 2022; Kim et al., 2023)
Cats	Immunocompetent	Highly susceptible; severe systemic disease with hyperinflammation, lymphoid pathology, and hemophagocytosis	Model of severe human SFTS; evaluation of immunomodulatory therapies targeting hyperinflammation	Genetic variability; biosafety concerns due to high viral shedding	(Park et al., 2019; Matsuu et al., 2023; Osako et al., 2024)
Dogs	Immunocompetent (or immunosuppressed)	Mild or asymptomatic infection; transient clinical signs under immunosuppression; non-lethal course	Studies of viral clearance, host resistance, and vaccine responses	Lack of severe disease limits evaluation of therapeutics for advanced pathology	(Ishijima et al., 2022; Park et al., 2022; Saga et al., 2023; Park et al., 2024)
Non-human primates	Immunocompetent	Mild, self-limiting infection; transient hematological changes; minimal pathology	Investigation of immune responses, viral kinetics, and preclinical safety	Failure to reproduce severe disease limits utility for efficacy studies	(Jin et al., 2015; Matsuno et al., 2017; Li et al., 2023)

IFNAR, interferon-α/β receptor; CNS, central nervous system; PBMCs, human peripheral blood mononuclear cells.

### 6.1 Rodent models

Rodents, particularly mice and hamsters, remain the foundation of DBV preclinical research due to their genetic tractability, reproducibility, and scalability. These models are well suited for mechanistic studies and high-throughput *in vivo* antiviral screening. However, their limited susceptibility to DBV infection in immunocompetent states and physiological differences from humans constrain their translational relevance.

#### 6.1.1 Mouse models

Immunocompetent wild-type mice are generally refractory to severe DBV infection. Following a high-dose challenge (10^5^ TCID_50_, strain SD4), Matsuno et al. (2017) observed neither fatal outcomes nor overt histopathological lesions across 12 wild-type strains. The observation that even aged C57BL/6 mice experience only transient weight loss highlights the efficiency of intact innate immunity in limiting DBV pathogenesis.

To bypass this restriction, researchers must disable type I interferon (IFN) signaling. Transient immune blockade via IFNAR-neutralizing antibodies is sufficient to induce susceptibility, resulting in hepatic necrosis, inflammation, and splenic white pulp atrophy (Park et al., 2020). However, genetic ablation of IFNAR provides a more uniformly lethal model. DBV-infected IFNAR^−/−^ C57BL/6 mice develop extreme viremia, severe thrombocytopenia, reticuloendothelial hyperplasia, and follicular destruction in cervical lymph nodes (Bryden et al., 2022). This clinical deterioration is driven by a cytokine storm involving IL-12, IL-6, IL-1α, IL-1β, and IFN-γ. Additionally, the model captures DBV neuroinvasion, where microglial activation drives the formation of neurotoxic A1 reactive astrocytes, ultimately causing neuronal death (Kim et al., 2024).

Given its reproducibility and severe disease profile, the IFNAR^−/−^ model serves as the primary platform for evaluating DBV countermeasures. It has been critical for defining the therapeutic dynamics of broad-spectrum antivirals like favipiravir and ribavirin (Tani et al., 2018). Today, the model is indispensable for the preclinical evaluation of novel small molecules, neutralizing antibodies (Huang et al., 2022; Shimojima et al., 2022; Xu et al., 2024), and the protective efficacy of emerging vaccine candidates (Hicks et al., 2024; Jeong et al., 2025).

While IFNAR^−/−^ mice are excellent for modeling systemic disease, their genetic immune deficiency can complicate the study of natural viral neurotropism. To address this, neonatal wild-type mice are frequently used; their incomplete immune maturation allows for severe central nervous system (CNS) infection without requiring genetic ablation. Unlike resistant adult mice, infected neonatal CD-1 mice develop overt neurological signs, including lethargy and tremors, 7–8 days post-infection (Liu et al., 2014). In neonatal BALB/c models, DBV directly targets microglia to trigger NLRP3 inflammasome activation, IL-1β secretion, and pyroptosis (Gao et al., 2022). Notably, intraperitoneal infection in neonatal C57BL/6 mice leads to rapid blood-brain barrier (BBB) penetration—reportedly within 3 hours—resulting in high brain viral loads and massive neuronal damage (Chen et al., 2022). Thus, neonatal models are uniquely valuable for assessing the BBB penetrance and neuroprotective efficacy of CNS-targeted antivirals.

Moving beyond viral dissemination and neurotropism, evaluating human-specific immunotherapies requires platforms with relevant immune targets. Since IFNAR^−/−^ and neonatal mice lack a mature, complete immune system, researchers have developed humanized mouse models to fill this niche. By engrafting human peripheral blood mononuclear cells (PBMCs) into immunodeficient NCG mice, Xu et al. (2021) created a HuPBL-NCG model that closely mimics clinical SFTS. Upon infection, these mice develop severe thrombocytopenia, leukopenia, cytokine storms, and multi-organ damage. Although donor-dependent engraftment variability remains a challenge, this system is uniquely suited for evaluating human-specific biologics, such as monoclonal antibodies and targeted cytokine inhibitors. Alternatively, investigating vaccine efficacy requires an intact murine immune system. Because wild-type mice resist infection, Park et al. (2023) circumvented this by delivering the human entry receptor DC-SIGN (hDC-SIGN) to wild-type C57BL/6 mice via an AAV2 vector. Following DBV challenge, these mice successfully exhibit platelet and leukocyte depletion. By increasing viral permissiveness without ablating native immunity, the hDC-SIGN model provides a more physiological environment for assessing vaccine protection and natural immune responses.

Despite these advances, each mouse model has inherent limitations. IFNAR-deficient models lack intact innate immune responses, restricting their use in studying immune-mediated pathogenesis or interferon-based therapies (Oh et al., 2024). Neonatal models are limited by immature immunity and rapid disease progression. Humanized systems, although promising, remain technically complex and variable. Future refinements could include improving the efficiency and consistency of human immune cell engraftment in humanized mice, developing models that retain more intact antiviral immune responses, and standardizing experimental conditions to improve reproducibility and physiological relevance.

#### 6.1.2 Hamster models

Syrian golden hamsters serve as a valuable alternative rodent model, offering distinct advantages for evaluating antiviral pharmacokinetics and viral pathogenesis. Consistent with wild-type mice, immunocompetent hamsters are refractory to DBV and exhibit no overt clinical disease (Jin et al., 2012; Liu et al., 2014). To establish a permissive model, Gowen et al. (2017) utilized a STAT2-knockout (STAT2^−/−^) hamster, which lacks intact type I and III interferon signaling. Following DBV challenge, these animals develop severe disease that closely recapitulates human pathogenesis, characterized by systemic inflammation, profound thrombocytopenia, elevated transaminases, and substantial viral dissemination localized primarily within hepatic and splenic tissues. Crucially, the STAT2^−/−^ hamster provides a highly discriminative platform for preclinical antiviral evaluation. Prophylactic or early therapeutic administration of favipiravir (T-705) conferred complete protection against lethal infection and significantly reduced tissue viral loads. In contrast, ribavirin failed to provide meaningful survival benefits or suppress viral replication (Gowen et al., 2017)—a differential response that aligns with the variable and often limited clinical efficacy of ribavirin in human SFTS cohorts. While this model is highly effective for screening direct-acting antivirals, its inherent interferon deficiency restricts its application for assessing immunomodulatory therapeutics. The future development of partially humanized or immune-reconstituted hamster models will be essential to further elucidate complex host–virus interactions and evaluate host-directed therapies.

### 6.2 Preclinical large animal models

Large animal models provide increased physiological and immunological similarity to humans and are therefore critical for translational research. Compared with rodent systems, these models are better suited for evaluating disease progression, immune responses, and therapeutic efficacy under clinically relevant conditions.

#### 6.2.1 Ferret models

Ferrets have emerged as a highly relevant immunocompetent model for DBV research. Unlike murine models that typically require genetic immunodeficiency (e.g., IFNAR^-/-^) to achieve lethality, ferrets naturally recapitulate the age-dependent pathogenesis observed in human SFTS cohorts. As demonstrated by Park et al. (2019), young adult ferrets (≤ 2 years of age) mount an effective antiviral response, exhibiting only mild, transient illness with complete survival. Conversely, aged ferrets (≥ 4 years of age) develop severe disease characterized by profound thrombocytopenia, leukopenia, sustained fever, extensive multi-organ viral dissemination, and up to 93% mortality. These findings resemble severe human SFTS, which is characterized by fever, thrombocytopenia, leukopenia, and potentially fatal multi-organ dysfunction, with older age associated with a higher risk of severe outcome (Chen et al., 2017; He et al., 2020). However, the high mortality observed under experimental challenge conditions may not reflect the overall case-fatality rate in human SFTS. Because it features a fully functional immune system, this age-stratified model is particularly suited for evaluating immunomodulatory therapies and host-directed antivirals, where an intact host immune response is a prerequisite for accurate efficacy assessment. Furthermore, the aged ferret model provides a rigorous platform for preclinical vaccine evaluation. For example, Kim et al. (2023) utilized this lethal challenge system to validate a self-assembling Gn head-ferritin nanoparticle vaccine, demonstrating that vaccination completely prevented clinical manifestations and conferred full protection in aged ferrets. As a viral envelope glycoprotein involved in host cell attachment and a target of neutralizing antibodies, Gn represents a promising vaccine antigen against DBV. The ferritin nanoparticle platform enables multivalent display of the Gn head region, potentially enhancing its immunogenicity (Kim et al., 2023). However, accurate pharmacological assessment requires strict standardization of experimental parameters. Park et al. (2022)) highlighted that the route of viral inoculation significantly alters disease kinetics and mortality. Intramuscular and subcutaneous challenges in aged ferrets induced rapid disease progression with 100% mortality, whereas intravascular inoculation resulted in a substantially lower mortality rate (25%) despite comparable early clinical signs. These distinct kinetic profiles emphasize the necessity of carefully calibrating infection routes when designing *in vivo* efficacy trials. Overall, by displaying severe clinical manifestations in an immunocompetent background, the aged ferret represents a robust translational model for advanced therapeutic and prophylactic evaluation.

#### 6.2.2 Companion animal models

Companion animals, especially cats and dogs, are of significant value in DBV research. Besides their epidemiological importance as intermediate hosts and direct sources of human infection (Kida et al., 2019; Yamanaka et al., 2020; Tsuru et al., 2021), these species show distinct susceptibilities to the virus. Such differential susceptibility serves as a reliable comparative system for studying viral pathogenesis and testing medical interventions.

Cats are highly susceptible to DBV infection, serving as a natural model for severe human SFTS. Both clinical and experimental evidence indicate that infected cats develop acute, lethal disease characterized by fever, severe cytopenia, weight loss, and hemophagocytosis (Park et al., 2019). Pathological investigations by Matsuu et al. (2023) revealed extensive necrotizing lymphadenitis and systemic viral dissemination across multiple body fluids. Because DBV consistently induces a lethal hyperinflammatory response in cats (Osako et al., 2024), this model is highly valuable for evaluating immunomodulatory therapies targeting the cytokine storm. Although the use of outbred feline populations introduces inter-breed variability that may complicate experimental reproducibility, it provides a genetically diverse background that better reflects human clinical settings.

In contrast, domestic dogs display relative resistance to DBV infection. While natural canine infections can result in mild clinical signs and detectable viral shedding (Ishijima et al., 2022; Saga et al., 2023), experimental challenge in immunocompetent dogs typically fails to cause overt morbidity. This relative resistance may be associated with effective host immune control. Park et al. (2022)) demonstrated that clinical symptoms, such as fever and weight loss, could only be induced under pharmacological immunosuppression (e.g., via azathioprine), yet the infection remained non-lethal. This immunosuppressed canine model is particularly useful for studying DBV infection in immunocompromised populations. Additionally, dogs serve as a relevant model for vaccine evaluation; for instance, administration of an inactivated DBV vaccine successfully cleared viremia and reduced splenic white pulp atrophy in challenged dogs (Park et al., 2024).

#### 6.2.3 NHP models

NHPs are highly relevant preclinical models due to their physiological and immunological similarities to humans. However, establishing a severe DBV infection model in immunocompetent macaques has proven challenging, as most species exhibit an inherent resistance to severe disease. This self-limiting clinical presentation currently restricts their utility for advanced therapeutic screening.

The relatively mild disease seen in immunocompetent macaques may reflect differences in host antiviral responses and viral replication dynamics. In rhesus macaques, DBV infection induces virus-specific IgM, IgG, and neutralizing antibodies, accompanied by changes in circulating immune cell populations and inflammatory cytokine levels (Jin et al., 2015; Li et al., 2023). Despite these responses, infected animals generally do not develop the severe systemic manifestations observed in fatal human SFTS cases. Notably, viral RNA can remain detectable in lymphoid tissues and bone marrow after viremia has become undetectable, suggesting that clinical recovery does not necessarily coincide with complete viral clearance (Jin et al., 2015). Differences in host susceptibility, viral strains, and experimental conditions may also influence disease outcomes. However, the mechanisms underlying the relative resistance of immunocompetent macaques to severe DBV infection remain unclear.

In Chinese rhesus macaques (Macaca mulatta), DBV inoculation predominantly results in a mild, transient clinical course. As reported by Li et al. (2023), infection elicits only low-grade fever, transient thrombocytopenia and leukopenia, alongside moderate elevations in transaminases and creatine kinase. Although middle-aged macaques (11–14 years) display a slight decline in coagulation function (Li et al., 2023), age-dependent disease exacerbation is minimal, with young adults (4–5 years) presenting similarly mild symptoms (Jin et al., 2015). Cynomolgus macaques (Macaca fascicularis) demonstrate an even higher threshold of resistance. Following high-dose viral challenge (10^6^ TCID_50_), clinical manifestations are negligible; infected animals show only isolated mild platelet reductions without overt histopathological lesions (Matsuno et al., 2017).

While wild-type NHP models are valuable for investigating viral clearance mechanisms and asymptomatic infections, their inability to recapitulate the lethal, hyperinflammatory pathogenesis of severe SFTS limits their efficacy in evaluating targeted interventions. Consequently, developing highly permissive NHP models remains a critical priority. Targeted disruption of type I interferon signaling, particularly IFNAR-mediated signaling, or carefully controlled pharmacological immunosuppression may represent potential strategies for increasing susceptibility to DBV infection in NHPs, based on evidence from interferon-impaired mouse models (Park et al., 2020; Bryden et al., 2022). Ultimately, such advanced *in vivo* platforms are essential for the robust validation of host-directed immunomodulators, therapeutics, and next-generation vaccines prior to clinical trials.

## 7 Future perspectives

Despite substantial progress in developing experimental models for DBV infection, no single system fully recapitulates the complexity of human SFTS. Key gaps remain in modeling severe inflammatory responses, age-dependent susceptibility, and multi-organ involvement. Addressing these limitations will be essential for improving the predictive value of preclinical studies and accelerating therapeutic development.

### 7.1 Future directions for NHP models

NHPs are widely regarded as the most clinically relevant preclinical models owing to their close immunological and physiological similarity to humans. However, currently available models consistently develop only mild or self-limiting disease following DBV infection and fail to reproduce the severe hyperinflammatory manifestations observed in fatal human cases.

Improving disease susceptibility in NHPs remains a critical objective. Targeted modulation of innate immune pathways, particularly type I interferon signaling, may offer a strategy to establish more permissive models. However, such approaches must be carefully balanced with ethical considerations and the need to maintain physiological relevance and model stability. Establishing a severe-disease NHP model would significantly boost the preclinical evaluation of antivirals and vaccine candidates.

### 7.2 Humanized and immunocompetent animal models

Interferon-deficient models, including IFNAR^−/−^ mice and STAT2^−/−^ hamsters, remain indispensable for antiviral efficacy studies due to their reproducible and often lethal phenotypes. However, the absence of intact interferon responses limits their utility for investigating immune-mediated pathogenesis and for evaluating immunomodulatory therapies.

Future efforts should focus on refining humanized mouse models with improved immune system reconstitution, enabling more accurate modeling of cytokine dysregulation and host–virus interactions. In parallel, immunocompetent models such as aged ferrets and domestic cats provide valuable systems for studying disease progression in the context of intact immunity. Reproducibility and cross-study comparison call for tighter standardization of key parameters (age, viral strain, inoculation route, dose).

### 7.3 One health considerations

Increasing evidence supports a role for companion animals in the ecology and transmission of DBV. Comparative studies between highly susceptible species, such as cats, and relatively resistant hosts, such as dogs, may help identify host determinants of disease severity and viral clearance.

Integration of veterinary and human research under a One Health framework may provide new insights into transmission dynamics and inform strategies to reduce zoonotic spillover. This could spur veterinary vaccines or other interventions that indirectly lower human infection risk.

### 7.4 Advanced *in vitro* platforms

Although conventional two-dimensional cell culture systems remain indispensable for mechanistic studies and antiviral screening, they fail to capture tissue architecture and multicellular interactions. This limitation is particularly relevant for DBV infection, which is driven by coordinated organ-specific injury rather than isolated cellular responses. Recent studies using human pancreatic organoids demonstrate that DBV exhibits defined tissue tropism and replicates within structured human tissues through a process mediated by host entry factors such as CCR2 and LRP1. Infection induces pronounced cell death alongside interferon-driven and pro-inflammatory responses, while transcriptomic analyses further reveal complement activation and inflammatory amplification not observed in monolayer systems (Liu et al., 2026). These findings indicate that three-dimensional models provide mechanistic resolution of tissue-specific host–virus interactions that cannot be achieved in reductionist systems. Incorporation of such platforms into preclinical pipelines would therefore enable more discriminative evaluation of candidate therapeutics prior to *in vivo* validation.

## 8 Conclusion

DBV research has reached a stage at which the central limitation is no longer the absence of experimental systems, but the mismatch between model capacity and experimental ambition. Existing *in vitro* and *in vivo* platforms each capture discrete components of SFTS pathogenesis, yet none fully reproduces the combined features of severe human disease. This is not merely a technical gap—it fundamentally shapes mechanistic inference and therapeutic assessment. The most informative path forward lies in deliberate model complementarity. Reductionist cell systems remain indispensable for defining viral and cellular mechanisms. Interferon-deficient rodents provide efficient platforms for establishing *in vivo* antiviral activity. Immunocompetent models such as aged ferrets and cats better reflect the inflammatory and clinical complexity of severe disease, while resistant hosts and non-human primates offer important insight into viral control and host restriction. The value of these models does not reside in their individual completeness, but in how they are deployed together. Accordingly, progress in the field will depend on a shift from model description to model strategy. Experimental systems must be selected according to the biological question being asked, and key findings should be validated across platforms with complementary strengths. Refinement of humanized, immunocompetent, and advanced multicellular models will further improve physiological fidelity, but methodological rigor and cross-model integration are equally important. A preclinical pipeline built on these principles will provide a stronger foundation for DBV antivirals, immunomodulatory therapies, and vaccines.
