# Supplementary material for: Preclinical models of Bandavirus dabieense infection: insights into pathogenesis, host responses, and translational applications
Source: Front Cell Infect Microbiol. 2026 Oct 29;16:2007854. doi: 10.3389/fcimb.2026.2007854 (PMC13756141; doi:10.3389/fcimb.2026.2007854)
Supplement: Supplementary file 1 [file Table1.docx]

***Supplementary Material***

**Supplementary Table 1. Search strategy in PubMed/MEDLINE**

| **Search number** | **Query** | **Results** |
| --- | --- | --- |
| #1 | "*Bandavirus dabieense*"[Title/Abstract] OR "Dabie bandavirus"[Title/Abstract] OR "severe fever with thrombocytopenia syndrome virus"[Title/Abstract] OR "severe fever with thrombocytopenia syndrome phlebovirus"[Title/Abstract] OR SFTSV[Title/Abstract] OR "severe fever with thrombocytopenia syndrome"[Title/Abstract] OR "SFTS virus"[Title/Abstract] OR "SFTS infection"[Title/Abstract] | 1808 |
| #2 | "cell model"[Title/Abstract] OR "cell models"[Title/Abstract] OR "cell line"[Title/Abstract] OR "cell lines"[Title/Abstract] OR Vero[Title/Abstract] OR "Vero E6"[Title/Abstract] OR HUVEC[Title/Abstract] OR HUVECs[Title/Abstract] OR macrophage*[Title/Abstract] OR organoid*[Title/Abstract] OR "animal model"[Title/Abstract] OR "animal models"[Title/Abstract] OR mouse[Title/Abstract] OR mice[Title/Abstract] OR murine[Title/Abstract] OR IFNAR[Title/Abstract] OR hamster*[Title/Abstract] OR STAT2[Title/Abstract] OR ferret*[Title/Abstract] OR cat[Title/Abstract] OR cats[Title/Abstract] OR feline[Title/Abstract] OR dog[Title/Abstract] OR dogs[Title/Abstract] OR canine[Title/Abstract] OR macaque*[Title/Abstract] OR "non-human primate"[Title/Abstract] OR "nonhuman primate"[Title/Abstract] OR "in vitro"[Title/Abstract] OR "in vivo"[Title/Abstract] OR preclinical[Title/Abstract] OR "experimental infection"[Title/Abstract] | 4911293 |
| #3 | #1 AND #2 | 464 |

Note: The database search was conducted on October 14, 2026.

**Supplementary Table 2. Search strategy in Web of Science Core Collection**

| **Search number** | **Query** | **Results** |
| --- | --- | --- |
| #1 | TS = ("*Bandavirus dabieense*" OR "Dabie bandavirus" OR "severe fever with thrombocytopenia syndrome virus" OR "severe fever with thrombocytopenia syndrome phlebovirus" OR SFTSV OR "severe fever with thrombocytopenia syndrome" OR "SFTS virus" OR "SFTS infection") | 1705 |
| #2 | TS = ("cell model" OR "cell models" OR "cell line" OR "cell lines" OR Vero OR "Vero E6" OR HUVEC OR HUVECs OR macrophage* OR organoid* OR "animal model" OR "animal models" OR mouse OR mice OR murine OR IFNAR OR hamster* OR STAT2 OR ferret* OR cat OR cats OR feline OR dog OR dogs OR canine OR macaque* OR "non-human primate" OR "nonhuman primate" OR "in vitro" OR "in vivo" OR preclinical OR "experimental infection") | 5352817 |
| #3 | #1 AND #2 | 464 |

Note: The database search was conducted on October 14, 2026.

**Supplementary Table 3. Summary of the retrospective structured literature search and evidence-reconciliation process**

| **Audit stage** | **Number of records/references** |
| --- | --- |
| Records identified from PubMed/MEDLINE | 464 |
| Records identified from Web of Science Core Collection | 464 |
| Total database records identified | 928 |
| Duplicate database records removed | 429 |
| Unique database records after deduplication | 499 |
| Additional references identified by reference-list or pre-existing-library reconciliation | 8 |
| Model-focused references captured by the structured database search | 51 |
| Model-focused references retained through manual or reference-list reconciliation | 8 |
| Total references cited in the final model-focused synthesis | 59 |

Note: This table summarizes a retrospective evidence audit performed during revision of a critical narrative review. It is intended to document the literature-identification and evidence-reconciliation process and should not be interpreted as a formal PRISMA study-selection flow. Full-text exclusion counts were not retrospectively reconstructed.
